# Quality of life in patients with statin intolerance: a multicentre prospective registry study

**DOI:** 10.1016/j.lanepe.2024.100981

**Published:** 2024-07-01

**Authors:** Paulina E. Stürzebecher, Ioanna Gouni-Berthold, Christina Mateev, Ole Frenzel, Stephan Erbe, Jes-Niels Boeckel, Markus Scholz, Ulrike Schatz, Oliver Weingärtner, Ursula Kassner, Ulrich Laufs, Statin Intolerance Registry investigators, A. Baessler, K. Borucki, G. Heine, G. Hoh, R. Klingenberg, W. Koenig, K. Parhofer, V. Rettig-Ewen, V. Schettler, S. Schirmer, S. Seiler-Mußler, K. Stach-Jablonski, J. Taggeselle, A. Tamm, A. Vogt

**Affiliations:** a Klinik und Poliklinik für Kardiologie, Universitätsklinikum Leipzig, Leipzig, Germany; b Center for Endocrinology, Diabetes and Preventive Medicine, University of Cologne, Faculty of Medicine and University Hospital Cologne, Cologne, Germany; c Institute for Medical Informatics, Statistics and Epidemiology, Leipzig, Germany; d LIFE Research Center for Civilization Diseases, University of Leipzig, Germany; e Department of Internal Medicine III, University Hospital Carl Gustav Carus, Technische Universität Dresden, Dresden, Germany; f University Hospital Jena, Department of Internal Medicine I, Jena, Germany; g Charité – University Medicine Berlin, Clinic for Endocrinology and Metabolic Medicine, Berlin, Germany

**Keywords:** Statin, Intolerance, Muscle, Symptoms, Pain, Women

## Abstract

**Background:**

Statin intolerance is associated with increased cardiovascular risk. Symptoms and patients’ characteristics are incompletely known. We aimed to analyse the health-related quality of life (QOL) associated with statin intolerance.

**Methods:**

The Statin Intolerance Registry (SIR) is an observational, prospective, multicentre study that included 1111 patients, with intolerance to at least two different statins, between 2021 and 2023 in Germany. SIR baseline data were compared to individuals with and without statin therapy of the population-based LIFE-Adult Study (n = 9983).

**Findings:**

The mean age in SIR was 66.1 years (standard deviation (SD) 9.9). The cohort was characterized by a higher proportion of women compared to patients on statins in LIFE-Adult (57.7% vs. 38.2%). SIR patients had impaired QOL (mean EQ VAS score of 64.9 (SD 18.1)) as measured by EuroQol (EQ-5D-5L)), which further deteriorated with age. Muscle symptoms were frequent (95.8%) and were associated with severe pain in 43.2% and intake of pain medication in 32.3% of statin intolerant patients. 10.3% had a diagnosis of depression. Women reported more pronounced symptoms than men. A data-driven k-means analysis, based on variables predicting severity of pain while on statin therapy, identified five clusters of SIR patients. The clusters differed in sex, prevalence of depression, QOL, comorbidities, and expectations to tolerate statin therapy.

**Interpretation:**

Statin intolerance is associated with impaired QOL. Women are more frequently and severely affected. The identified clusters may help to identify patients at risk and to develop individualized strategies to improve patient trajectories and outcomes.

**Funding:**

10.13039/501100008678Leipzig University, research grants from Daiichi Sankyo, Novartis, and Amgen to 10.13039/501100008678Leipzig University.

**Research in context**

**Evidence before this study**

There is a large body of evidence supporting the widespread use of statins for reducing atherosclerotic cardiovascular disease. However, statin intolerance affects a significant number of patients and is associated with increased cardiovascular risk. Patient characteristics, risk factors and quality of life related to statin intolerance are largely unknown. We conducted a comprehensive search on PubMed until April 16, 2024 using the search terms (statin intolerance OR statin-associated muscle symptoms OR SAMS), (statin) AND (pain OR quality of life OR QOL OR hrQOL). The search revealed only limited evidence regarding quality of life of patients with statin intolerance or quantitative data on the intensity of pain intensity while on statin therapy. Female sex has been shown as risk factor for the development on of statin intolerance. However, sex differences regarding quality of life, symptoms of depression and pain in statin intolerant patients are unknown. Previous publications are based on meta-analysis or small patient numbers.

**Added value of this study**

To our knowledge, this is the first prospective, contemporary, multicentre registry investigating clinical characteristics and quality of life of patients with statin intolerance. The study included 1111 patients between 2021 and 2023. In comparison to a cohort representative of the general population, a diagnosis of hypothyroidism or depression is more prevalent in statin intolerant patients. Patients with statin intolerance have a highly impaired quality of life. Women are more frequently and more severely affected. They are suffering from higher intensity of muscle pain during statin therapy than men and report more severely impaired daily activity due to statin-associated muscle pain. Depressive symptoms are consistently more prevalent in women in comparison to men across all age groups. Five clusters of patient characteristics were identified that differed in age, sex, comorbidities, quality of life and lifestyle. The cluster analysis suggests a heterogeneous etiology of statin intolerance. The observed data complements the evidence from meta-analysis and indicate a high unmet medical need to better identify, prevent and to provide individualized treatment for this condition.

**Implications of all the available evidence**

Statin intolerance affects a high number of patients and is associated with elevated cardiovascular risk. From the patient perspective, statin intolerance is associated with a highly decreased quality of life. Special attention should be paid to women, as they are more frequently and severely affected. The data reveal clusters of possible different etiologies of statin intolerance. This provides the opportunity to improve patient trajectories by providing individualized strategies to prevent and to treat statin intolerance.

## Introduction

Cholesterol-lowering by 3-Hydroxy-3-Methylglutaryl-Coenzym-A (HMG-CoA) reductase inhibitors, statins, reduces the risk of atherosclerotic cardiovascular disease.^1^ Statins are therefore among the most widely used drugs worldwide. Multiple high-quality studies have demonstrated the excellent long-term efficacy, safety, and tolerability of statins.^2^ However, a significant number of patients are unable to tolerate guideline-recommended doses of statins, a condition denoted as “statin intolerance”.^3^, ^4^, ^5^ The most frequently reported adverse events are statin-associated muscle symptoms (SAMS).^5^ Statin intolerance is associated with primary and secondary non-adherence, discontinuation of treatment, insufficient cholesterol lowering, increased atherosclerotic cardiovascular disease and higher mortality.^6^^,^^7^ Important aspects of the pathogenesis, diagnosis, prognosis, and potential treatment options are unknown despite the clinical importance of statin intolerance.^5^^,^^8^ Due to these uncertainties, slightly different definitions of statin intolerance are used e.g. by the European Atherosclerosis Society (EAS), the National Lipid Association (NLA), the Luso-Latin American Consortium (LLAC) or the Canadian Consensus Working Group (CCWG).^3^^,^^5^^,^^9^^,^^10^ A large meta-analysis of 176 studies with >4 million patients reported an overall prevalence rate of 9.1% which was largely independent of the definition applied or the type of statin used.^3^ Therefore, several millions of patients worldwide are reporting statin intolerance.

According to a widely accepted definition of statin intolerance that was used in the large CLEAR Outcomes trial, statin intolerant patients are defined as the ones being unable or unwilling to receive statins owing to an adverse effect that had started or increased during statin therapy and resolved or improved after statin therapy was discontinued.^11^ This and all other definitions of statin intolerance are primarily based on the symptoms that are reported by patients. Therefore, a better understanding of the perception of pain and the quality of life of patients with statin intolerance is needed. The characteristics of patients with statin intolerance and their symptoms remain incompletely understood but are of key importance to adequately address the open questions related to this common condition.

The prospective, multicentre, observational Statin Intolerance Registry (SIR) recruited patients in Germany with intolerance to at least two different statins. Symptoms had to disappear or improve after discontinuation of statin therapy. The aim of this baseline analysis is to gain a better understanding of the patient characteristics and the health-related quality of life (HRQOL) of patients with statin intolerance compared to the general population and to individuals on statin therapy. Despite the high prevalence of statin intolerance, the underlying pathologies are incompletely understood, making the diagnosis and management of statin intolerance difficult. Therefore, we aimed to identify clusters of patients within the population of patients with statin intolerance to assess the underlying aetiologies as potential basis for tailored strategies.

## Methods

### Study design of the statin intolerance registry

The statin intolerance registry (SIR) is a prospective, observational, non-interventional, multicentre study. It enrolled 1111 patients presenting to outpatient lipid clinics (cardiology and nephrology medical practices and university lipid clinics) in Germany from May 2021 to June 2023 at the 19 participating sites (Supplemental Figure S1, Table S1). Patients were recruited consecutively. Each local Ethics Committee approved the study according to their respective regulations. The study is conducted in accordance with the Declaration of Helsinki and Good Clinical Practice. Written informed consent was obtained from all participants before data collection. In this longitudinal study, data will be collected prospectively at annual visits. The study was registered at ClinicalTrials.gov under NCT04975594. The sample size for this observational study was determined based on the anticipated prevalence of the condition of interest and the desired precision of the estimated association between variables of interest and statin intolerance.

### Study population and data of the statin intolerance registry

Patients were eligible for the study if they had dyslipidaemia and statin intolerance. Statin intolerance was defined as intolerance of 2 or more statins either in any dose or the inability to tolerate an increase in the statin dose beyond a maximum intake of 70 mg atorvastatin, 140 mg simvastatin, pravastatin, or lovastatin, 35 mg rosuvastatin, 280 mg of fluvastatin per week. Moreover, symptoms had to improve or disappear when the statin dose was reduced or discontinued.^11^^,^^12^ Participants had to be 18 years or older, give written informed consent and be capable to meet the study requirements. The study excluded patients who had used any experimental or investigational drugs within 30 days prior to screening as well as employees or contractors of the facilities conducting the study, or family members of the principal investigator, co-investigator, or sponsor.

This non-interventional study documented data based on standard of care. Diagnosis and treatment were decided by the attending physician. Data collected at baseline included demographic parameters, comorbidities, details of former statin therapies and associated adverse events, laboratory parameters as well as current treatment. Comorbidities were assessed via medical records for SIR patients. In the LIFE-Adult study comorbidities were documented based on self-reported statements or current treating therapies.

In addition, standardized questionnaires regarding depression (PHQ-9) and health-related quality of life measured by EuroQol (EQ-5D-5L) were completed by SIR patients. Study data were collected and managed using REDCap electronic data capture tools hosted at the Center for Clinical Studies (ZKS), Leipzig, Germany.^13^

The PHQ-9 questionnaire is a 9-item survey using 3-point response scales (0—not at all, 3 nearly every day), which measures depressive symptoms in the past 2 weeks based on the Diagnostic and Statistical Manual of Mental Disorders, 5th edition (DSM-5) criteria for a major depressive episode. The scores of the items are added up to a total score, ranging from 0 to 27.^14^ A score of 5–10 indicates mild depressive symptoms while a score of ≥10 is indicative of moderate to severe depressive symptoms. The EQ-5D-5L questionnaire was used to measure health related quality of life. It consists of a visual analogue scale (EQ VAS) ranging from 0 (worst imaginable health state) to 100 (best imaginable health state). Furthermore, it consists of five questions assessing the current health state with regard to five dimensions: mobility, self-care, usual activities, pain/discomfort, and anxiety/depression.^15^ The EQ-5D-5L health index is a summary of the EQ-5D-5L health state which considers the preferences of the general population of a country, in this case of Germany as proposed by EuroQOL.^16^

Muscle pain while on statin therapy was assessed by a numeric rating scale (NRS) ranging from 0 to 10 with 0 representing “no pain” and 10 “unbearable pain.”^17^ The interference with general activity due to statin-associated muscle pain was also measured on a scale ranging from 0 to 10, with 10 representing complete interference and 0 no interference with activities of everyday life (adapted from the Brief Pain inventory questionnaire^18^). The statin associated muscle symptom clinical score (SAMS-CI) was assessed for patients reporting muscle pain while on statin therapy. According to Rosenson et al. a score of <7 indicates that the muscle symptoms are most likely not associated with statin therapy.^19^ A score of 7 or 8 indicates a possible association, while a score of 9 or higher represents a probable association between muscle symptoms and statin therapy.

Experience of negative statin-related news was reported based on individual assessments. Patients were asked to report whether they expected to tolerate the statin well before the first prescription. Cardiovascular (CV) risk was assessed based on patient data and using the CV classification according to the 2019 ESC/EAS guidelines.^1^ Comorbidity score based on ICD-10 codes was quantified according to Stausberg et al.^20^

### LIFE-adult cohort

Participants who were enrolled in the population-based adult cohort of the “Leipzig Research Centre for Civilization Diseases” (LIFE) served as controls to our SIR cohort.^21^ Adult residents of the German city of Leipzig aged 18–80 years were invited randomly for study participation using the population registry.^22^ Patients with missing data regarding lipid-lowering medication were excluded from the analysis (*n* = 17). Subgroups of patients with statin treatment (*n* = 1284, named “LIFE_Statin”) respectively without statin treatment (*n* = 8699, named “LIFE_NoStatin”) were defined. The LIFE-Adult population was described in detail elsewhere.^21^

### Data analysis

Baseline characteristics are presented as mean (standard deviation [SD]) of continuous variables and as frequencies and percentages of categorical variables. Student's *t*-test was applied for the analysis of group differences of continuous variables. Chi-squared test was used to compare binary variables between groups. One-way ANOVA and Chi-squared Omnibus t were used to compare more than two groups. Bonferroni correction was applied to control for multiple testing.

To balance the baseline characteristics between the SIR cohort and LIFE-Adult cohort, we matched cases (SIR patients) and controls (LIFE-Adult individuals) by sex and age (1-year band). One control was randomly matched with one case.^23^ Patients for whom no control was found were excluded from the analysis (n = 64). McNemar's test was used to assess differences between SIR patients and matched controls.

Spearman correlation was calculated to test for correlation of pain intensity and pain related interference of general activity. Multiple linear regression was applied to evaluate predictors of EQ VAS score. For this analysis multiple imputation was conducted by using chained equations for five imputations. A total of 16 variables based on clinical experience were chosen for analysis (age; sex; BMI; high education; married; smoking; premature CV event; diabetes; comorbid score (as described above); NRS pain intensity while on statin therapy; SAMS-CI score; muscle pain at study inclusion; report of negative statin-related news from family, friends, media, pharmacy, medical staff; PHQ-9 score; currently on lipid lowering therapy, physically active). Logistic regression did not suggest violation of the missing at random (MAR) or missing completely at random (MCAR) assumptions. Missing values for these variables were <8%, except for NRS pain intensity while on statin therapy (10% of missingness) and the SAMS-CI score (13% missing variables).

Statistical analyses were performed using Stata/MP 18.0. No imputation was carried out for missing values apart from k-means cluster analysis and multiple linear regression analysis and missing values were not considered when calculating relative frequencies.

The most relevant variables for muscle pain and impairment of everyday activities due to muscle pain while on statin therapy were identified using the random forest algorithm. The random forest model was configured with a selection threshold of 85% and utilized 500 trees (ntrees = 500) using the RandomForest R pacakge.^24^ The six most relevant variables out of 36 (basic characteristics (age, BMI), depression (PHQ-9 score, diagnosis of depression), health related quality of life (EQ VAS score, score in EQ-5D-5L pain dimension), comorbidities (atherosclerotic cardiovascular, hypertension, diabetes, active smoking, CV event, premature CV event or revascularization, hypothyroidism, muscle disease, chronic renal failure, sleep apnoea, steatosis hepatis, atrial fibrillation, rheumatic disease, orthopaedic disease, cancer, heart failure, chronic obstructive lung disease), statin associated muscle symptoms (SAMS-CI score, use of pain medication, expectation of side effects while on statin therapy, side effects while on statin not affecting the musculature, CK elevation while on statin, elevated liver enzymes while on statin, negative statin related news on media, acquaintances with statin intolerance, negative statin related news by treating physicians/pharmacy/family/friends), psycho-social (in a relationship, physically active, high education)) were age, BMI, PHQ-9 score, EQ VAS, EQ dimension on pain/discomfort and SAMS-CI score. Missing values for these variables were <8%, except for the SAMS-CI score where 13% of the values were missing. Median imputation was used for missing values. K-means clustering was done using the k-means function in the cluster package^25^, ^26^, ^27^, ^28^ of the statistical software package R version 4.1.2. The elbow method and gap statistics were used to identify the optimal number of clusters. Visualization of clusters was performed by t-distributed stochastic neighbour embedding (t-SNE).^29^

### Role of the funding source

This study was funded by Leipzig University and research grants from Daiichi Sankyo, Novartis, and Amgen to Leipzig University. The funding sources had no part in study design, data collection, data analysis, data interpretation, or writing of the report.

## Results

A total of 1111 patients with statin intolerance were included in the baseline analysis of the SIR (Supplemental Figure S1). Patients' demographics, characteristics, and comorbidities were compared to the population-based LIFE-Adult-Study (*n* = 9983). In LIFE-Adult, 1284 individuals were on statin, while 8699 participants did not report statin medication. Patients’ characteristics are detailed in Table 1.

**Table 1:** Comparison of baseline characteristics between the SIR- and the LIFE-Adult populations.

	SIR (*n* = 1111)	LIFE_NoStatin (*n* = 8699)	LIFE_Statin (*n* = 1284)	*P-*value (SIR vs. LIFE_NoStatin)	*P-*value (SIR vs. LIFE_Statin)
**Demographic data**					
Age (years), mean ± SD	66.1 ± 9.9	56.1 ± 12.3	66.7 ± 8.7	<0.001^a^	0.096^a^
Female, %	57.7	54.4	38.2	0.04^b^	<0.001^b^
Systolic blood pressure (mmHg), mean ± SD	140.1 ± 18.4	128.3 ± 16.8	130.8 ± 18.0	<0.001^a^	<0.001^a^
Diastolic blood pressure (mmHg), mean ± SD	81.9 ± 10.4	75.7 ± 9.8	73.0 ± 10.1	<0.001^a^	<0.001^a^
BMI (kg/qm), mean ± SD	27.4 ± 4.6	27.0 ± 4.8	29.4 ± 4.9	0.03^a^	<0.001^a^
**Laboratory parameters**					
Total cholesterol (mmol/L), mean ± SD	4.8 ± 1.7	5.7 ± 1.0	4.9 ± 1.0	<0.001^a^	0.095^a^
LDL (mmol/L), mean ± SD	2.8 ± 1.5	3.6 ± 0.9	2.9 ± 0.9	<0.001^a^	0.02^a^
HDL (mmol/L), mean ± SD	1.5 ± 0.5	1.6 ± 0.5	1.5 ± 0.4	<0.001^a^	0.99^a^
Triglycerides (mmol/L), mean ± SD	1.8 ± 1.1	1.3 ± 1.0	1.7 ± 1.3	<0.001^a^	0.19^a^
Creatinine (μmol/L), mean ± SD	88.1 ± 53.0	78.8 ± 15,4	88.8 ± 42.2	0.30^a^	0.98^a^
**Cardiovascular risk factors**					
Hypertension, %	75.2	33.5	72.8	<0.001^b^	0.29^b^
Diabetes, %	19.1	10.2	39.3	<0.0001^b^	<0.001^b^
Packyears, mean ± SD	24.2 ± 19.2	16.3 ± 17.8	22.8 ± 21.4	<0.001^b^	0.26^b^
Atherosclerotic cardiovascular disease, %	88.0	39.8	74.5	<0.001^b^	<0.001^b^
Coronary heart disease, %	65.4	1.1	22.4	<0.001^b^	<0.001^b^
Peripheral artery disease, %	53.3	39.3	69.6	<0.001^b^	<0.001^b^
Myocardial infarction, %	31.2	0.7	15.0	<0.001^b^	<0.001^b^
Stroke, %	9.9	1.3	8.2	<0.001^b^	0.14^b^
Arterial revascularization procedures, %	50.2	1.8	26.3	<0.001^b^	<0.001^b^
**Comorbidities**					
Hypothyroidism, %	23.3	14.7	18.5	<0.001^b^	0.003^b^
Joint arthrosis, %	18.8	22.5	33.6	0.006^b^	<0.001^b^
Cancer, %	10.9	3.2	6.9	<0.001^b^	0.001^b^
Depression, %	10.3	4.9	4.4	<0.001^b^	<0.001^b^
Pollen allergy, %	9.6	9.5	6.3	0.88^b^	0.003^b^
Heart failure, %	7.6	0.8	4.2	<0.001^b^	<0.001^b^
Asthma, %	7.3	4.2	6.0	<0.001^b^	0.20^b^
Rheumatic disease, %	4.9	4.4	7.8	0.46^b^	0.004^b^
Chronic obstructive pulmonary disease %	4.1	6.0	8.6	0.011^b^	<0.001^b^

Patients included in the statin intolerance registry (SIR) were compared to the population-based LIFE -Adult cohort. LIFE-Adult participants were stratified for statin use (LIFE_NoStatin vs. LIFE_Statin). Demographic data, lipid parameters, cardiovascular risk factors and comorbidities are presented.

BMI, body mass index; HDL, high density lipoprotein; LDL, low density lipoprotein; SD, standard deviation.

a *t*-test, adjusted significance level after Bonferroni correction *P* < 0.006.

b χ^2^ test, adjusted significance level after Bonferroni correction *P* < 0.003.

The mean age of SIR patients was 66.1 years (SD 9.9) and 57.7% were women. In comparison, 38.2% of the LIFE-Adult participants on statin therapy were women. Most of the patients enrolled in the SIR had very high cardiovascular risk and 88.0% had documented atherosclerotic cardiovascular disease. The prevalence of orthopaedic diseases was not higher in SIR in comparison to the LIFE-Adult populations. Significantly more patients in SIR had hypothyroidism compared to LIFE-Adult, while values of thyroid-stimulating hormone were similar (1.12 mU/L vs. 1.17 mU/L, *P =* 0.96) (Table 1). Stratification and matching for age and sex confirmed the higher prevalence of depression and hypothyroidism in patients with statin intolerance compared to the general population (Supplemental Figure S2A–C). A total of 26.9% of statin intolerant patients was on statin therapy at study inclusion (27.2% of statin intolerant women, 26.4% of statin intolerant men).

### Quality of life

**Fig. 1:**
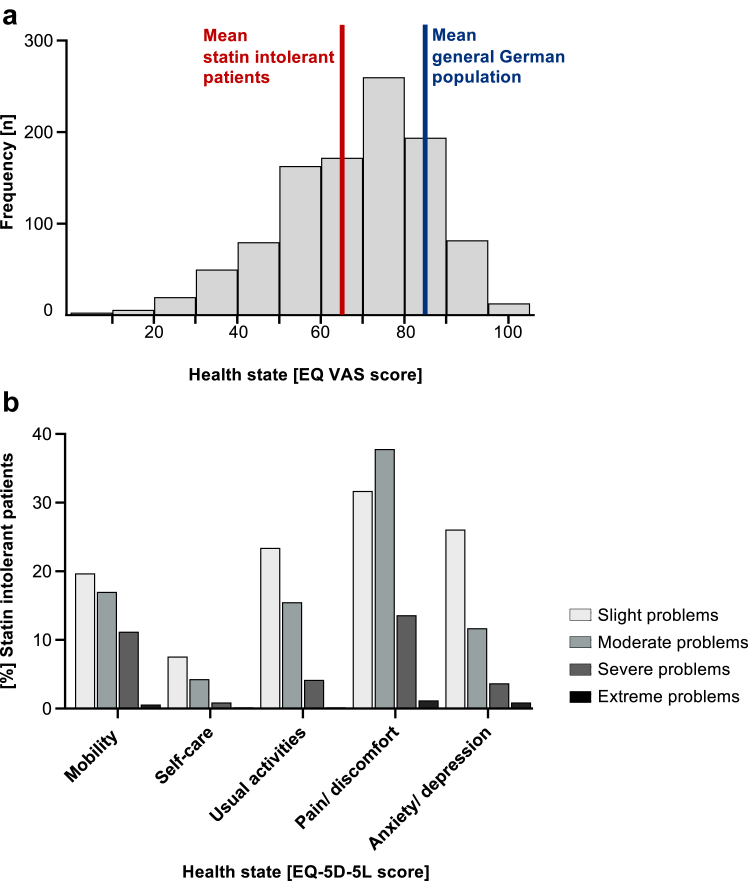
**Depressive symptoms and health related quality of life in patients with statin intolerance**. (a) EQ-5D-5L VAS frequency distribution in the SIR cohort (*n* = 1037). Red line shows the mean of 64.9 in SIR patients. The blue line represents the mean of 85.1 in the general German population.^20^ (b) Distribution of responses to the EQ-5D-5L descriptive system which comprises the following five dimensions: Mobility, self-care, usual activities, pain/discomfort, and anxiety/depression). Each dimension has five response levels. The graph shows level 2 (slight problems) to level 5 (extreme problems). SIR, Statin Intolerance Registry.

The overall quality of life in SIR patients was markedly reduced. EQ VAS scores, which quantifies a person's general health status, were ranging from 0 to 100, indicating a high intra-individual variability in HRQOL in statin intolerant patients. The mean EQ VAS score was 64.9 (SD 18.1) (Table 2, Fig. 1a). For comparison, the mean EQ VAS score in the general German population in 2015 was 85.1.^30^ At enrolment, only 10.9% of all SIR patients stated no problems in the five EQ-5D-5L dimensions. The most frequently reported EQ-5D-5L dimension with moderate, severe, or extreme symptoms was pain/discomfort (55.0%), followed by mobility (32.6%), usual activity (23.9%), and anxiety/depression (20.4%). Problems in self-care (9.9%) were less frequent (Fig. 1b). Multiple linear regression analysis revealed that age, female sex, higher BMI, premature CV event or revascularization, lower PHQ-9 scores as well as physical inactivity and muscle pain at enrolment were associated with reduced HRQOL (Table 3). Mean EQ VAS scores of SIR patients currently taking a statin were similar in comparison to SIR patients currently not on statin therapy (64.2 vs. 66.6, *P* = 0.06).

**Table 2:** Quality of life stratified by sex and age groups.

Age	<55 yrs	55–66 yrs	67–79 yrs	>80 yrs	All
	n = 126	n = 418	n = 469	n = 98	n = 1111
**EQ VAS**					
All	67.4 (20.0)	64.9 (18.2)	64.8 (17.2)	61.8 (19.4)	64.9 (18.1)
Male	68.0 (18.9)	66.6 (17.4)	67.4 (19.3)	68.2 (19.4)	67.3 (17.7)
Female	66.5 (21.8)	63.4 (18.8)	63.3 (17.0)	56.6 (17.9)	63.1 (18.2)
**EQ-5D-5L index**					
All	0.82 (0.23)	0.79 (0.23)	0.79 (0.22)	0.75 (0.22)	0.79 (0.23)
Male	0.81 (0.24)	0.82 (0.21)	0.83 (0.19)	0.86 (0.14)	0.82 (0.20)
Female	0.82 (0.22)	0.77 (0.24)	0.77 (0.23)	0.67 (0.24)	0.76 (0.24)
**PHQ-9 score**					
All	5.8 (4.8)	6.3 (4.6)	5.4 (4.3)	5.7 (3.7)	5.8 (4.4)
Male	5.1 (4.2)	5.9 (4.4)	4.6 (4.0)	4.7 (3.6)	5.3 (4.2)
Female	6.9 (5.5)	6.5 (4.7)	5.8 (4.4)	6.4 (3.7)	6.1 (4.5)

Age groups were based on 10th, 50th, and 90th percentile. Mean number of a visual analogue scale for self-rated health state (EQ VAS), the mean EQ-5D-5L index, which is calculated by deducting the appropriate German specific weights according to Ludwig.^15^ as well as mean PHQ-9 scores, indicating depression, are shown for sex, age groups and total SIR cohort, respectively. Standard deviations are shown in parentheses. Yrs, years.

**Table 3:** Multiple linear regression analysis^a^ on self-reported health-related quality of life (EQ VAS score) in statin intolerant patients.

Parameter	Estimate	Standard error	t-value	Pr > |t|	95% confidence interval	
Age (years)	−0.25	0.05	−5.1	**<0.001**	−0.36	−0.16
Female sex	−2.18	1.06	−2.05	**0.04**	−4.29	−0.72
BMI (kg/qm)	−0.18	0.11	−1.7	**0.001**	−0.40	0.03
High education^b^	−0.6	1.16	−0.54	0.59	−2.90	1.64
Married	1.76	0.99	1.78	0.08	−0.18	3.70
Current smoker	−1.02	1.57	−0.65	0.51	−4.10	2.05
Premature CV event or revascularization	−4.36	1.13	−3.85	**<0.001**	−6.60	−2.14
Diabetes	−2.63	−1.26	−2.09	0.04	−5.11	−0.15
Comorbid score *(ranging from 89 to 111 in integers)*	0.13	0.17	0.75	0.46	−0.22	0.48
NRS pain intensity while on statin *(ranging from 0 to 10, in integers)*	−0.13	0.30	0.45	0.66	−0.71	0.45
SAMS-CI score *(ranging from 0 to 11, in integers)*	−0.28	0.25	−1.07	0.29	−0.78	0.23
Muscle pain at enrollment	−5.20	1.03	−5.09	**<0.001**	−7.29	−3.20
Report of negative statin-related news from family, friends, media, pharmacy, medical staff	−0.64	0.97	−0.66	0.51	−2.54	1.27
PHQ-9 score *(ranging from 0 to 27, in integers)*	−1.72	0.12	−14.43	**<0.001**	−1.95	−1.48
Currently on lipid lowering therapy	0.56	1.28	0.44	0.66	−1.96	3.09
Not physically active	−6.40	1.27	−5.03	**<0.001**	−8.90	−3.90

BMI, body mass index; CV, cardiovascular; NRS, numeric rating scale; SAMS-CI, statin associated muscle symptoms clinical index.

a R^2^ = 0.33, R^2^ adj = 0.31.

b High education defined as university degree. A comorbidity score based on the structure of the ICD-10 was calculated according to Stausberg.^19^

### Depressive symptoms

Diagnosis of depression was more frequent in SIR than in LIFE-Adult participants (10.3% vs. 4.8%) (Table 1). The overall mean PHQ-9 score in SIR was 5.8 (SD 4.4) (Table 2). Measured by PHQ-9 questionnaire, mild signs of depressive symptoms were prevalent in 33.9% of SIR patients while 23.1% showed signs of moderate to severe depression. The percentage of LIFE-Adult participants with depressive symptoms either on a statin or not was much lower (LIFE_Statin 18.5% vs. LIFE_NoStatin 14.3%, CES-D score ≥22). Patients with signs of moderate to severe depression reported a significantly higher intensity of muscle pain while on statin therapy (NRS pain scale 7.3 vs. 6.9, *P =* 0.006). No significant difference in PHQ-9 scores was observed in patients on statin therapy in comparison to patients without statin therapy (5.5 vs. 5.9, *P* = 0.17).

### Pain

**Fig. 2:**
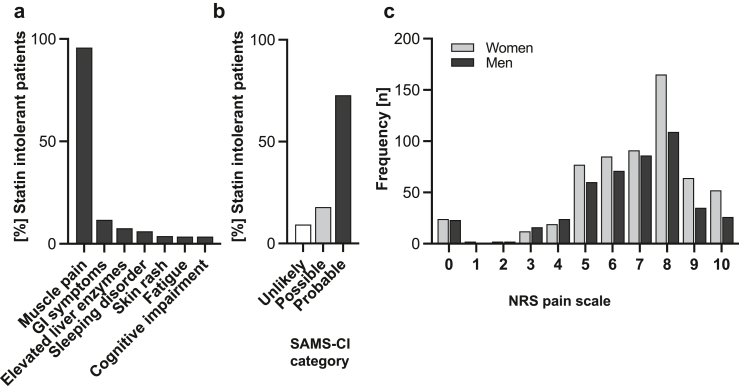
**Adverse effects and pain in patients with statin intolerance**. (a) Prevalence of the seven most common reported adverse effects while on statin therapy. (b) Frequency of SAMS-CI score according to Rosenson et al.^21^ (*n* = 971 of 1095 patients with statin associated muscle symptoms available). (c) Intensity of muscle pain while on statin therapy measured by numeric rating scale (NRS), stratified by sex (*n* = 998 of 1095 patients with SAMS available). GI, gastro-intestinal; NRS, numeric rating scale; SAMS-CI, SAMS, statin associated muscle symptoms; SAMS clinical score.

Muscle pain was the most frequently reported side effect while on statin therapy (95.8%) (Fig. 2a). The Statin-Associated Muscle Symptom Clinical Index (SAMS-CI) was used as a method to assess the likelihood that a patient's muscle symptoms were associated with statin use.^19^ 72% of patients reported SAMS-CI scores above 9 points indicating that the muscle symptoms were probable caused or worsened by statin use (Fig. 2b).

The intensity of muscle pain while on statin therapy was assessed using the NRS pain scale (mean NRS score: 7.0/10.0, SD 1.8). 43.2% of patients reported a score of 8 or above indicating severe pain. 31.9% experienced moderate pain (NRS 6–7 points) while on statin therapy (Fig. 2C). The intensity of statin-associated muscle pain correlated with severity of interference with general activity (r = 0.63, 95% CI 0.59–0.67*, P* < 0.001). 32.3% of the patients with statin intolerance used pain medication because of SAMS.

15.6% of patients with statin intolerance had elevation of creatin kinase (CK) while on statin therapy. In the SIR cohort, 4 patients (2 women, 2 men) had a history of rhabdomyolysis. CK elevation was not associated with more intense SAMS (NRS pain scale: mean 6.7 vs. 7.1, *P =* 0.04) nor with more physical activity (>4 days/week of physical activity 14.5% vs. 15.6%, *P =* 0.75).

At the time of enrolment, 46.6% of SIR patients reported to have muscle pain (NRS pain scale: mean 5.4, SD 2.3). The intensity of muscle pain at enrolment correlated with the PHQ-9 score (r = 0.27, 95% CI 0.21–0.33, *P* < 0.001), the EQ VAS (r = −0.30, 95% CI −0.36 to −0.25, *P* < 0.001), and the EQ-5D-5L index (r = −0.46, 95% CI −0.51 to −0.41, *P* < 0.001).

### Sex differences

Women were more frequently and more severely affected by statin intolerance. Women had lower EQ VAS and EQ-5D-5L index scores when compared with their male counterparts (Table 2). The observed effects were consistent across all five EQ-5D-5L dimensions (Supplemental Table S2). Linear regression analysis on EQ VAS revealed female sex as a strong predictor for reduced HRQOL (Table 3). Women reported higher levels of pain affecting health related quality of life than men (mean score in EQ-5D-5L pain dimension: 2.7 vs. 2.4, *P* < 0.001). Women had lower quality of life compared to men in all sub-categories.

Women with statin intolerance were more likely to show major depressive symptoms compared to men with statin intolerance (25.8% vs. 19.4%, *P* = 0.01). PHQ-9 scores were higher in women in comparison to men in each age group (Table 2). While on statin therapy, women reported a higher intensity of muscle pain (NRS 7.1 vs. 6.8, *P =* 0.003) (Fig. 2C) and higher interference with general activity than men (NRS 6.7 vs. 6.3, *P =* 0.02). Women were less likely to experience CK elevation while on statin therapy (9.5% vs. 23.6%, *P* < 0.001).

### Age effects

The percentage of patients with moderate, severe, or extreme problems increased over age in every of the five descriptive EQ-5D-5L dimensions (Supplemental Table S2). Furthermore, EQ VAS and EQ-5D-5L index scores diminished with increasing age, especially in women (Table 2). Age was a significant predictor for lower HRQOL in the linear multiple regression analysis (Table 3).

### Cluster analysis

**Fig. 3:**
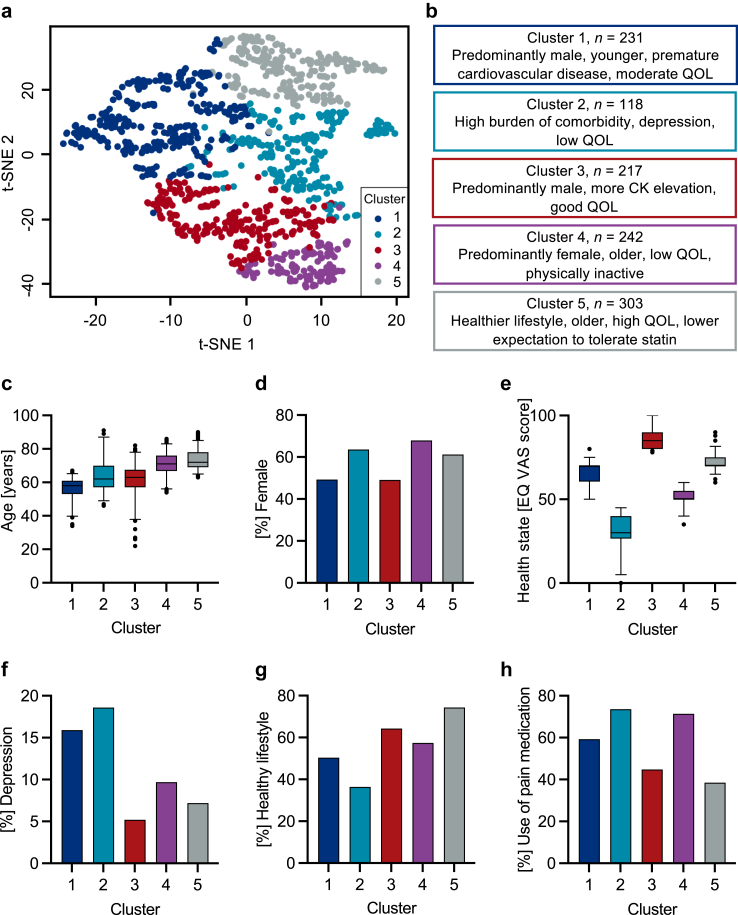
**Clusters of patient characteristics in patients with statin intolerance**. (a) K-means cluster analysis identified five clusters that are grouped according to the six most relevant variables for predicting severity of pain while on statin therapy. T-distributed stochastic neighbour embedding (t-SNE) was used for visualization. (b) Description of identified clusters by most distinctive characteristics. (c) Distribution of age, (d) sex, (e) health related quality of life (EQ VAS score), (f) prevalence of depression, ((g) prevalence of individuals with healthy lifestyle (defined as non-obese, non-smoking, physically active)), and (h) use of pain medication due to SAMS for each cluster. BMI, body mass index; EQ VAS, EQ5D5L visual analogue scale on health state; QOL, quality of life; SAMS, statin associated muscle symptoms.

A cluster analysis of the SIR patients was performed to better understand the underlying patterns and differences within the population. Data driven analysis identified the six most relevant variables for the prediction of pain intensity and interference with general activity due to muscle pain while on statin therapy, namely age, BMI, PHQ-9 score, EQ VAS, EQ dimension on pain/discomfort and SAMS-CI score. Based on these variables, k-means clustering identified five clusters of patients with statin intolerance (Fig. 3a and b).

Cluster 1, including 231 patients, consisted predominantly of males, relatively young patients with a high prevalence of premature cardiovascular events or need for arterial revascularization. The HRQOL was moderately reduced (EQ VAS 66.7, SD 7.2) and diagnosis of depression was highly prevalent (15.8%, mean PHQ-9 score of 6.1 (SD 4.1)) (Fig. 3c–h, Table 4). Cluster 2 patients (*n* = 118) were characterized by a high burden of comorbidities in comparison to the other clusters, a high prevalence of depression (18.6%) and a highly reduced HRQOL (EQ VAS 31.0, SD 6.0). The mean PQH-9 score indicated moderate to severe major depressive symptoms (10.7, SD 5.5) (Fig. 3c–h). Cluster 3 included 217 predominantly male patients with a very good quality of life, few comorbidities expect atherosclerotic cardiovascular event, mostly in longterm relationship. There was a tendency for more elevations of CK and liver enzymes due to statin therapy (Fig. 3c–h, Table 4). Cluster 4 (*n* = 242) was characterized by predominantly older, female patients with low quality of life and more chronic kidney disease in comparison to cluster 1 and 3. Fewer individuals of this cluster reported being in a committed stable relationship, and a high percentage was not engaging in sports (Fig. 3c–h, Table 4). Cluster 5 (*n* = 303) was characterized by a healthy lifestyle defined as no smoking, no obesity, physically active. Patients in cluster 5 had the lowest percentage of participants with the initial expectation to tolerate the statin (Fig. 3c–h, Table 4). As internal control, the cluster analysis was repeated separately for men and women obtaining qualitatively comparable results. In summary, the most important differences between the clusters related to sex, age, quality of life, depressive symptoms, comorbidities, and lifestyle (Fig. 3, Table 4).

**Table 4:** Cluster analysis of patient characteristics.

	Cluster 1: Predominantly male, younger, premature ASCVD, moderate QOL	Cluster 2: High burden of comorbidity, depression, low QOL	Cluster 3: Predominantly male, good QOL	Cluster 4: Predominantly female, older, low QOL, physically inactive	Cluster 5: Healthier lifestyle, older, high QOL, lowest expectation to tolerate statin	*P*-value
	n = 231	n = 118	n = 217	n = 242	n = 303	
**Demographic data**						
Age (years), mean ± SD	56.5 ± 6.6	63.9 ± 9.4	61.7 ± 9.2	71.0 ± 6.9	73.4 ± 5.8	<0.001^b^
Female, %	49.3	63.6	49.1	67.9	61.2	<0.001^c^
BMI (kg/qm), mean ± SD	28.4 ± 5.4	28.4 ± 5.0	26.9 ± 4.3	27.7 ± 4.8	26.1 ± 3.6	<0.001^b^
**Laboratory parameter**						
LDL (mmol/L), mean ± SD	2.8 ± 1.5	3.0 ± 1.6	2.8 ± 1.5	2.8 ± 1.6	2.8 ± 1.5	0.64^b^
**Cardiovascular risk factors**						
Hypertension, %	59.9	83.9	69.5	82.3	80.1	<0.001^c^
Diabetes, %	15.0	26.3	14.8	24.5	18.2	0.01^c^
Active Smokers, %	14.5	14.4	9.1	11.0	4.5	0.001^c^
Atherosclerotic cardiovascular disease, %	81.9	89.8	85.7	89.0	93.5	<0.001^c^
CV event, %	42.7	50.9	35.2	47.7	46.6	0.03^c^
Premature cv event or art. revascularization, %	46.3	39.8	25.7	23.2	12.3	<0.001^c^
**Comorbidities**						
Hypothyroidism, %	23.8	20.3	18.6	24.1	27.7	0.17^c^
Orthopedic disease, %	42.3	62.7	35.2	56.5	48.6	<0.001^c^
Cancer, %	7.9	12.7	5.2	14.4	12.7	0.01^c^
Depression, %	15.9	18.6	5.2	9.7	7.2	<0.001^c^
Chronic kidney disease, %	4.0	11.9	9.1	19.0	15.1	<0.001^c^
Heart failure, %	4.4	10.2	4.3	10.1	8.5	0.03^c^
Rheumatic disease, %	10.1	11.9	5.2	11.4	7.9	0.12^c^
Chronic obstructive pulmonary disease, %	2.6	10.2	1.0	8.0	2.1	<0.001^c^
**Psycho-social**						
EQ VAS score, mean ± SD	66.7 ± 7.2	31.0 ± 9.3	86.3 ± 6.0	51.6 ± 5.9	73.1 ± 6.0	<0.001^b^
PHQ-9 score, mean ± SD	6.1 ± 4.1	10.7 ± 5.5	3.3 ± 2.7	6.7 ± 3.8	4.6 ± 3.7	<0.001^b^
In longterm relationship, %	73.1	72.9	83.8	67.5	72.6	0.003^c^
High education^a^, %	18.1	16.1	22.4	19.4	25.0	0.18^c^
Healthy lifestyle, %	50.4	36.4	64.3	57.4	74.4	<0.001
No physical activity, %	15.0	37.3	12.4	19.8	7.2	<0.001^c^
**Other parameters of interest**						
CK elevation while on statin, %	18.5	15.3	18.1	13.1	13.0	0.27^c^
Elevated liver enzymes while on statin, %	9.3	7.6	11.0	3.8	6.9	0.05^c^
Intensity of muscle pain while on statin, mean ± SD	7.0 ± 1.6	7.3 ± 1.7	6.7 ± 2.0	7.1 ± 1.7	7.0 ± 1.8	0.03^b^
Use of pain medication due to SAMS, %	59.3	73.6	44.8	71.4	38.5	<0.001^c^
Negative statin-related reports from family, friends, media, pharmacy, medical staff, %	36.6	31.4	36.2	38.8	41.1	0.42^c^
Expectation to tolerate statin, %	67.3	78.0	71.9	65.0	63.1	0.03^c^

The data driven k-means cluster analysis revealed five clusters. Patient characteristics are displayed for each cluster regarding demographic data, LDL cholesterol, cardiovascular risk factors, comorbidities, psycho-social characteristics (mean number of a visual analog scale for self-rated health state (EQ VAS), and PHQ-9 score) and details on statin side effects and pain while on statin therapy.

Art., arterial; BMI, body mass index; CK, creatine kinase; CV, cardiovascular; LDL, low density lipoprotein; QOL, quality of life assessed by EQ-5D-5L questionnaire; SD, standard deviation; SAMS, statin associated muscle symptoms.

a High education defined as university degree.

b *t*-test, adjusted significance level after Bonferroni correction *P* < 0.007.

c χ^2^ test, adjusted significance level after Bonferroni correction *P* < 0.003.

## Discussion

Statin intolerance is a common phenomenon with important clinical implications. However, the aetiology, diagnosis, prognosis, and treatment strategies of statin intolerance are incompletely defined and understood and therefore a matter of scientific debate.^3^, ^4^, ^5^^,^^8^ To our knowledge, the Statin Intolerance Registry is the largest database to date that prospectively collected detailed characteristics, comorbidities, health related quality of life and depressive symptoms of patients with statin intolerance. The baseline analysis of the SIR revealed several important findings: Patients with statin intolerance have a highly impaired quality of life and high levels of pain. Among patients with statin intolerance, women are more frequently and more severely affected. The identified clusters show that within the wide spectrum of patients with statin intolerance, subgroups can be identified. This information points towards a heterogeneous aetiology of statin intolerance and suggests for the first time that different types of statin intolerance may benefit from targeted prevention and treatment strategies.

Patients with statin intolerance reported a broad spectrum of HRQOL that is overall markedly reduced. The mean EQ VAS score in the SIR (64.9) is 20.1 points lower compared to the general German population.^30^ The EQ VAS score in statin intolerant patients is similar to the quality of life of survivors of myocardial infarction (EQ VAS score 65.8).^31^ When considering age, an analysis of the elderly population (aged 65–93 years with a mean age of 73.1 years (SD 5.7)) in Germany revealed a mean EQ VAS score of 73.2 (SD 18.5) which is still 8.3 points higher than in our cohort of statin intolerant patients with a mean age of 66.1 years.^32^ Only one in ten statin intolerant patients did not have any complaints in the five EQ-5D-5L dimensions at enrolment. Every second patient with statin intolerance had signs of depression. A diagnosis of depression in statin intolerant patients was significantly more common compared to patients on statin in the LIFE-Adult cohort. The prevalence of pain in statin intolerance is high (55.0%). The mean pain intensity on the numeric rating scale during statin therapy was ≥7/10 indicating severe pain. Orthopaedic comorbidities were similar in statin intolerant patients and the LIFE-Adult cohort. The mean SAMS-score was 9.0. These data confirm that most of the reported muscle pain in the SIR was associated with statin intake. Only a small proportion of patients (15.6%) was experiencing creatin kinase (CK) elevations while on statin therapy and those did not report increased severity of pain, which is in line with the current literature.^5^ Related to pain, every third patient reported problems of mobility and every fourth patient was impaired in usual activities while problems of self-care were infrequent. One in three patients with statin intolerance used pain medication due to SAMS. Taken together, patients with statin intolerance have a markedly reduced quality of life with frequent and significant pain. From the patient's perspective, these data indicate a high unmet medical need to better identify, prevent and treat this condition.

The SIR indicates that the prevalence of statin intolerance is higher in women than in men. 57.7% of the SIR population were female. In comparison, in the LIFE-Adult-Study only 38.2% of participants on statin therapy were females. This finding is in agreement with the literature,^33^ e.g. the large meta-analysis by Bytyci et al.^3^ and the CLEAR outcomes trial.^11^ Reasons for this result are unknown and may be related to biological, hormonal and genetic factors.^34^ A novel finding of the prospective SIR was that not only the frequency but also the extent of impairment is higher in women. Women with statin intolerance had poorer HRQOL as indicated by reduced EQ VAS and EQ-5D-5L index scores than men with statin intolerance. The observed effects were consistent across all five EQ-5D-5L dimensions. Furthermore, women were suffering from higher intensity of muscle pain during statin therapy than men and felt more severely impaired in their daily activity due to SAMS. Depressive symptoms were consistently more prevalent in women in comparison to men across all age groups. These findings are supported by data from the existing literature on pain susceptibility.^35^, ^36^, ^37^ Taken together, the data identify a sex difference in statin intolerance.

Another important risk marker for impaired HRQOL in statin intolerant patients is age. The percentage with moderate, severe, or extreme problems increased with age in all dimensions.

Additional risk markers identified in the multiple regression analysis include premature atherosclerotic cardiovascular disease, obesity, pre-existing muscle symptoms and depressive symptoms. Hypothyroidism was more frequent in comparison to the general population, a finding that has been reported previously.^3^^,^^38^ The higher prevalence of hypothyroidism was confirmed by stratification and matching for sex and age. The identified markers for impaired HRQOL can be easily recognized in primary care settings and may therefore help to identify patients at risk for statin intolerance.

Statin therapy in the large randomized controlled statin trials and the cholesterol treatment trialist (CTT) analyses caused only a small excess of mostly mild muscle pain.^4^^,^^39^ The majority of muscle symptoms (∼90%) represent most likely nocebo effects.^3^^,^^4^^,^^40^ The prevalence of statin intolerance is higher in observational studies and a recent large meta-analysis (∼10%).^33^ Statin intolerance is a heterogenous syndrome which underlying reasons are incompletely understood. Clinical management of statin intolerance can be difficult as no simple diagnostic test exists. In the light of the severe impairment of life reported by the patients with statin intolerance, there is a high unmet need for improved risk assessment and management. The size and the deep phenotyping of the SIR population provided the opportunity to perform an unbiased data-driven cluster analysis to explore underlying aetiologies as potential basis for tailored strategies. The analysis identified five clusters of patients. The main differences between the clusters related to age, sex, HRQOL, depressive symptoms, comorbidities, and lifestyle. These analyses allow several conclusions. First, the population of patients with statin intolerance encompasses different subgroups that have distinct phenotypes and most likely specific aetiologies. Second, the prevention and treatment strategies for statin intolerance likely need to be individualized. For example, individuals with healthy lifestyle that were exposed to negative reports on statin-associated problems and had lower expectations to tolerate the statin from the beginning on (cluster 5) may benefit from strategies that focus on information and education that differ from strategies for patients who have a diagnosis of depression and many comorbidities (cluster 2). Men with CK elevations, relatively high quality of life, and few comorbidities (predominantly in cluster 3) may primarily need non-statin lipid lowering drugs while other subgroups, e.g. elderly women that are physically inactive (cluster 4), may benefit from strategies that focus on physical activity and other lifestyle changes. These data set the stage to prospectively test individualized trajectories. In addition, the follow-up of the SIR will provide information regarding the clinical trajectories of the different clusters.

The registry recruited patients in cardiology and nephrology medical practices and university lipid clinics in Germany. Potential selection bias of recruiting in these settings include a higher number of patients with atherosclerotic cardiovascular disease and potentially more severely affected individuals. Limitations of the SIR and the population-based LIFE-Adult cohort relate to the predominantly Caucasian population and the potential selection bias of including individuals willing to participate in an observational study. Furthermore, while SIR was a multicentre study including patients in 19 different study cites in 17 Germany cities, the LIFE-Adult cohort was recruited primarily in Leipzig, Germany, and surroundings. The inclusion criteria of the SIR were somewhat more stringent compared to the widely accepted definition of statin intolerance used in the CLEAR outcomes trial.^11^ The difficulties defining statin intolerance have been discussed elsewhere.^5^ We believe that the definition of the SIR included a patient population that is highly clinically relevant. The follow-up of the SIR may contribute to improving the understanding of the aetiologies and thereby the definition of statin intolerance in the future. The use of validated questionnaires for depression (PHQ-9), health related quality of life (EQ-5D-5L), muscle pain (NRS scale) and the SAMS-CI score for statin associated muscle symptoms is an important strength of this study.

### Conclusions

In conclusion, the SIR provides quantitative information on the severely impaired quality of life in patients presenting with statin intolerance. Within this population, women appear to be more frequently and more severely affected. The cluster analysis suggests a heterogeneous aetiology of statin intolerance that may be helpful for defining future tailored treatment strategies.

## Contributors

UL and PES conceived the study. UL secured funding. PES, SE, CM performed the analyses. These authors as well as the last author had full data access. PES and UL drafted the paper. SE, CM, IGB, OF, JNB, MS, US, OW, UK provided comments on the paper and participated in data interpretation. All authors made the decision to submit the manuscript for publication. All authors meet the International Committee of Medical Journal Editors criteria for authorship for this article and have given their approval for this version to be published.

## Data sharing statement

Post-publication, deidentified data is available for other researchers through reasonable scientific request to the corresponding author with a signed data access agreement fulfilling the legal and regulatory requirements, and with approval from the German Ethical Review Authority.

## Declaration of interests

PES has received honoraria from Daiichi Sankyo, Amgen, Novartis, Synlab and from Novartis for an advisory board. IGB has received honoraria from Amgen, Regeneron, Sanofi, Aegereon, Akcea Therapeutics, Novartis, Daiichi-Sankyo, Synlab, Ultragenyx, and Amarin. MS received funding from Pfizer Inc., and Owkin for projects not related to this research. US has received honoraria from Amgen, Amarin, AstraZeneca, Boehringer Ingelheim, Daiichi Sankyo, Sanofi, Synlab, Novartis, NovoNordisk. OW reports honoraria for lectures from AMGEN, Berlin-Chemie, Daiichi-Sankyo, Novo Nordisk, Novartis, Sanofi-Aventis, Fresenius, Hexal, Akcea Therapeutics, and honoraria for advisory board activities from AMGEN, Sanofi-Aventis, Berlin-Chemie, Novartis, Hexal, Akcea Therapeutics, Daiichi-Sankyo, Pfizer, and Sobi. UK has received honoraria from Daiichi Sankyo, Amgen, MSD, Synlab. UL has received honoraria from Amgen, AstraZeneca, Bayer, Boehringer, Daiichi-Sankyo, Lilly, MSD, Novartis, NovoNordisk, Pfizer, Sanofi, Synlab. CM, OF, SE, JNB have nothing to declare.
