# Supplementary material for: Quality of life in patients with statin intolerance: a multicentre prospective registry study
Source: Lancet Reg Health Eur. 2024 Jul 1;43:100981. doi: 10.1016/j.lanepe.2024.100981 (PMC11263645; doi:10.1016/j.lanepe.2024.100981)
Supplement: Supplementary Figures and Tables [file mmc1.docx]

**Supplementary Material**

**Quality of life in patients with statin intolerance: a multicentre prospective registry study**

Paulina E. Stürzebecher^a^, Ioanna Gouni-Berthold^b^, Christina Mateev^a^, Ole Frenzel^a^, Stephan Erbe^a^, Jes-Niels Boeckel^a^, Markus Scholz^c,d^, Ulrike Schatz^e^, Oliver Weingärtner^f^, Ursula Kassner^g^, Ulrich Laufs^a^

on behalf of the Statin Intolerance Registry investigators

*^a^ Klinik und Poliklinik für Kardiologie, Universitätsklinikum Leipzig, Leipzig, Germany*

*^b^ Center for Endocrinology, Diabetes and Preventive Medicine, University of Cologne, Faculty of Medicine and University Hospital Cologne, Cologne, Germany*

*^c^ Institute for Medical Informatics, Statistics and Epidemiology, Leipzig, Germany*

*^d.^ LIFE Research Center for Civilization Diseases, University of Leipzig, Germany*

*^e^ Department of Internal Medicine III, University Hospital Carl Gustav Carus, Technische Universität Dresden, Dresden Germany*

*^f^ University Hospital Jena, Department of Internal Medicine I, Jena Germany*

*^g^ Charité – University Medicine Berlin, Clinic for Endocrinology and Metabolic Medicine, Berlin, Germany*

# Address for correspondence:

Dr. med. Paulina E. Stürzebecher

Klinik und Poliklinik für Kardiologie

Universitätsklinikum Leipzig

Liebigstr. 20

04103 Leipzig

Tel. Sekretariat:          +49-341-97 12650

Fax Sekretariat:         +49-341-97 12659

[Paulina.Stuerzebecher@medizin.uni-leipzig.de](mailto:Paulina.Stuerzebecher@medizin.uni-leipzig.de)

**Figure S1: Flow chart of the recruitment of the SIR cohort.**

**Figure S2: Prevalence of comorbidities in SIR vs LIFE-Adult cohort stratified by age and sex.** (A) Prevalence of hypothyroidism and (B) depression in SIR and LIFE-Adult individuals stratified for sex and age groups < 50 years (SIR: women n= 20, men n= 31; LIFE-Adult: women n= 1,700, men n= 1,461), 50-60 years (SIR: women n= 101, men n= 118; LIFE-Adult: women n= 1,240, men n= 1,051 ), 60-70 years (SIR: women n= 242, men n= 163; LIFE-Adult: women n= 1334, men n= 1,207) and > 70 years (SIR: women 277, men n=158; LIFE-Adult: women n=950, men n= 1,040). Chi-squared test, * P < 0.05. (C) Prevalence of five relevant comorbidities of statin intolerant patients vs. age -and sex-matched controls (LIFE-Adult cohort) (n= 1,047 per group). Odds ratio determined by McNemar’s test. A total of 64 patients were excluded from the analysis as no matching with the LIFE-Adult cohort was possible.

**Table S1: List of 19 participating study sites.**

| **Study site** | **Principal investigator** |
| --- | --- |
| University Hospital Regensburg | A. Baessler |
| University Hospital Magdeburg | K. Borucki |
| University Hospital Cologne | I. Gouni-Berthold |
| Agaplesion Markus Hospital Frankfurt am Main | G. Heine |
| Outpatient clinic Lutherstadt/Wittenberg (“Kardiologie-Wittenberg“) | G. Hoh/ A. Tamm |
| Charité University Hospital, Berlin | U. Kassner |
| Kerkoff Hospital Bad Nauheim | R. Klingenberg |
| Munich Heart Center | W. Koenig |
| University Hospital Leipzig | U. Laufs |
| University Hospital Munich (Campus Großhadern) | K. Parhofer |
| Outpatient clinic “Schwemlinger Gemeinschaftspraxis” | V. Rettig-Ewen |
| University Hospital Carl Gustav Carus, Dresden | U. Schatz |
| Göttingen outpatient clinic “Nephrologisches Zentrum Göttingen Gbr” | V. Schettler |
| Kaiserslautern outpatient clinic “Kardiopraxis Schirmer” | S. Schirmer |
| Saarlouis outpatient clinic “Vauban Praxis” | S. Seiler-Mußler |
| University Hospital Mannheim | K. Stach-Jablonski |
| Outpatient clinic Markkleeberg | J. Taggeselle |
| University Hospital Munich (Campus Innenstadt) | A. Vogt |
| Jena University Hospital | O. Weingärtner |

**Table S2:** **Percentage of patients with moderate, severe, or extreme problems in five descriptive EQ-5D-5L dimensions stratified by sex and age.**

| **EQ-5D-5L dimensions** | **Age groups** | | | | **Total** |
| --- | --- | --- | --- | --- | --- |
|  | **<55 yrs** | **55-66 yrs** | **67-79 yrs** | **>80 yrs** |  |
|  | *n* = 126 | *n* = 418 | *n* = 469 | *n* = 98 | *n* = 1111 |
|  | % | % | % | % | % |
| **Mobility** |  |  |  |  |  |
| All | 21.4 | 28.2 | 34.8 | 51.5 | 32.3 |
| Male | 18.7 | 23.9 | 30.1 | 47.7 | 27.5 |
| Female | 25.5 | 31.7 | 37.3 | 55.8 | 35.6 |
| **Self-care** |  |  |  |  |  |
| All | 6.4 | 9.1 | 9.8 | 18.2 | 9.9 |
| Male | 6.7 | 7.5 | 7.4 | 13.6 | 7.9 |
| Female | 5.9 | 10.4 | 11.1 | 18.9 | 11.1 |
| **Usual activities** |  |  |  |  |  |
| All | 27.8 | 21.3 | 23.0 | 34.3 | 23.9 |
| Male | 28.0 | 18.6 | 18.4 | 22.7 | 20.4 |
| Female | 27.5 | 23.5 | 25.5 | 41.5 | 26.3 |
| **Pain/ discomfort** |  |  |  |  |  |
| All | 45.2 | 54.8 | 55.7 | 65.7 | 55.0 |
| Male | 48.0 | 47.3 | 49.1 | 54.6 | 48.7 |
| Female | 41.2 | 50.9 | 59.2 | 73.6 | 59.5 |
| **Anxiety/ depression** |  |  |  |  |  |
| All | 19.8 | 21.5 | 17.7 | 29.3 | 20.4 |
| Male | 16.0 | 19.2 | 11.0 | 27.3 | 16.6 |
| Female | 25.5 | 23.5 | 21.2 | 28.3 | 23.0 |

Age groups were based on 10^th^ 50^th^ and 90^th^ percentile. Percentage of patients with level 3-5 per descriptive domain of EQ5D5L questionnaire are presented for each age group as well as males and females separately. Yrs, years;
